# Resolving taxonomic uncertainties in the genus *Haemophilus*: a genomics-based approach for the reclassification of species within genera of the family *Pasteurellaceae* and proposal of four novel genera and one novel species

**DOI:** 10.3389/fmicb.2026.1798515

**Published:** 2026-03-31

**Authors:** Guillem Seguí, Beatriz Piñeiro-Iglesias, Francisco Salvà-Serra, Roger Karlsson, Edward R. B. Moore

**Affiliations:** 1 Department of Clinical Microbiology, Sahlgrenska University Hospital, Gothenburg, Sweden; 2 Culture Collection University of Gothenburg (CCUG), Sahlgrenska University Hospital and Sahlgrenska Academy, University of Gothenburg, Gothenburg, Sweden; 3 Department of Infectious Diseases, Institute of Biomedicine, Sahlgrenska Academy, University of Gothenburg, Gothenburg, Sweden; 4 Unit of Medical Technology and Biological Function, Department of Medical Technology and Diagnostics, RISE Research Institutes of Sweden, Mölndal, Sweden; 5 Nanoxis Consulting AB, Gothenburg, Sweden

**Keywords:** *Haemophilus* species, novel genera, novel species, *Pasteurellaceae*, phylogenomics, reclassification, taxonomy

## Abstract

**Introduction:**

The taxonomy of species within the genus *Haemophilus*, within the family *Pasteurellaceae*, shows extensive polyphyly, causing long-standing taxonomic ambiguities that complicate clinical, epidemiological and evolutionary research. Traditional classifications based on phenotypes and comparative 16S rRNA gene sequence analyses have resulted in discrepancies in defining genera and species, necessitating a thorough genome-based reevaluation. In this study, we have conducted an integrated phylogenomic analysis of the type strains of 90 species, covering all genera with validly published names within the family *Pasteurellaceae*, focusing on the historically-problematic *Haemophilus* genus.

**Methods:**

We used whole-genome sequencing, core proteome phylogeny and genome-wide similarity analyses to assess and define genus and species boundaries.

**Results:**

Results from phylogenomic analyses identified four well-supported species-clades within *Haemophilus*, revealing several misclassified species. Comparative POCP and AAI analyses, using genomic sequence data, showed that traditional genus-level thresholds (≥50% for POCP and 60–80% for AAI) of calculated protein content are insufficient to resolve species of genera with extensive horizontal gene transfer, whereas more stringent cutoffs aligned better with phylogenomic groupings. ANI and dDDH analyses effectively delineated species-level boundaries but offered limited detail for higher taxonomic ranks. Analyses of virulence factors found conserved sets of core genes known to be crucial for colonization, immune evasion and iron uptake, along with genus- and species-specific factors, indicating ecological adaptations. Functional annotation and metabolic pathway analysis highlighted universal processes and phylogenetic lineage-specific features.

**Discussion:**

Overall, our comprehensive genomic approach has elucidated a reliable phylogenetic-based taxonomy of *Haemophilus*, detected misclassified species, recognized new genera and supports a biologically meaningful taxonomy for *Pasteurellaceae*. These results establish the basis for accurate species identification, clinical diagnostics, evolutionary research and functional studies within this medically and veterinarily important family.

## Introduction

The genus *Haemophilus* (Winslow et al., 1917; Kilian, 2015; Nørskov-Lauritsen, 2021), within the family *Pasteurellaceae*, consists of Gram-negative, facultatively anaerobic coccobacilli and exemplifies particular taxonomic challenges. *Haemophilus* spp. include a broad range of bacteria that are considered part of the normal microbiota, as well as potential pathogens that infect humans and animals (Kuhnert and Christensen, 2008; Kilian, 1976). The genus *Haemophilus* exhibits extensive polyphyletic branching in DNA sequence-based phylogenetic analyses and does not form coherent clusters of species (Christensen and Bisgaard, 2018) as the *Pasteurella* and *Actinobacillus* species do within *Pasteurellaceae* (Naushad et al., 2015). This taxonomic complexity has led to debates over species delineations (Nørskov-Lauritsen, 2014). The genus *Haemophilus* comprises 15 species with validly published names (last accessed January 2026)^1^: *H. aegyptius, H. ducreyi* (Christensen and Kuhnert, 2012); *H. felis, H. haemolyticus, H. influenzae, H. massiliensis, H. paracuniculus, H. parahaemolyticus, H. parainfluenzae, H. paraphrohaemolyticus, H. piscium, H. pittmaniae, H. seminalis,* and *H. sputorum* (Nørskov-Lauritsen et al., 2012). Among these, at least 10 species have been reported to affect humans, with six species being particularly clinically-significant: *H. aegyptius, H. ducreyi, H. haemolyticus, H. parahaemolyticus, H. parainfluenzae,* and *H. influenzae. H. influenzae* (Lehmann and Neumann 1896; Winslow et al., 1917) is the most commonly observed species of *Haemophilus* linked to human disease, causing a wide variety of infections (Khattak and Anjum, 2023; Sethi et al., 2008; Casey and Pichichero, 2004; Peltola, 2000). Other species names exist as synonyms or have been effectively published but not validated (List of Prokaryotic names with Standing in Nomenclature, LPSN) (Parte et al., 2020).

Inconsistencies in the taxonomy of *Haemophilus* are reflected in the limitations of the traditional classification of *Haemophilus* spp. Despite the growing knowledge about *Haemophilus* diversity, many questions regarding its evolutionary dynamics and functional variability have remained unanswered (Bixler et al., 2025; Zhang et al., 2024; Carrera-Salinas et al., 2021). Historically, the species of *Haemophilus* have been classified mainly based on phenotypic characterizations, clinical manifestation and limited molecular techniques (Musher, 1996). However, the advent of advanced genomic methods have proven to be insufficient for reliable classification of *Haemophilus* spp. (Bianconi et al., 2023; Nørskov-Lauritsen, 2014). Phylogenomic-based studies increasingly show *Haemophilus* to be paraphyletic or even polyphyletic. This indicates that the genus, as classically defined, either excludes some descendant lineages or contains species that do not share an exclusive common ancestor, indicating that the current taxonomy does not reflect the evolutionary relationships among the species (Liang et al., 2021; Christensen et al., 2007; Rokas and Holland, 2000). Horizontal gene transfer (HGT) appears to be frequent within *Pasteurellaceae* and contributes to observed genomic mosaicism and polyphyly, complicating phylogenetic inference and highlighting the need for genome-scale analyses to resolve evolutionary relationships (da Silva et al., 2022; Hegstad et al., 2020; Michael et al., 2018; Nielsen et al., 2015; Kelley et al., 2007; Redfield et al., 2006).

The family *Pasteurellaceae* (Pohl, 1981), within the order *Pasteurellales*, includes a diverse range of Gram-negative, non-spore-forming, facultatively anaerobic bacteria, many of which are opportunistic pathogens. Members of this family can cause a wide range of localized infections and disseminated systemic infections in both humans and animals, including respiratory tract infections, urinary tract infections, septicemia and meningitis (Michael et al., 2018; Duell et al., 2016; Christensen and Kuhnert, 2012; Spinola et al., 2002). Given their medical and veterinary impact, a clear and robust taxonomic framework is essential. Traditional classifications of species of genera within the family have been based mainly on phenotypic traits, including morphology, growth factor requirements (hemin and NAD, Nicotinamide Adenine Dinucleotide) and biochemical profiles. While these approaches provided initial taxonomic frameworks, they have proven insufficient for defining phylogenetically coherent groups, due to extensive phenotypic variability and frequent genotypic convergence among unrelated lineages (Blackall and Turni, 2025; Christensen et al., 2014, 2015, 2020a; Nørskov-Lauritsen, 2014, 2021; Harris et al., 2020; Dewhirst et al., 1992; Kilian, 2015).

An accurate taxonomy of the family *Pasteurellaceae* is essential for reliable clinical diagnostics, epidemiological surveillance and antimicrobial therapy. Misclassification and ambiguous identification can lead to poor understanding of pathogen impact, transmission and, ultimately, incorrect treatment plans. Therefore, resolving taxonomic inconsistencies within *Pasteurellaceae* is vital, not only from a systematic point of view but also for applied microbiology and public health (Bisgaard and Christensen, 2025; Christensen et al., 2020b; Nørskov-Lauritsen, 2014; Bisgaard, 1995; MacInnes and Borr, 1990).

These challenges have been addressed through relatively new molecular and genomic approaches, leading to multiple taxonomic revisions across *Pasteurellaceae* (Christensen and Bisgaard, 2018). Several species previously classified within *Haemophilus* have been reassigned to other genera to better reflect their phylogenetic relationships. For example, *H. actinomycetemcomitans* was reclassified as *Aggregatibacter actinomycetemcomitans* (Nørskov-Lauritsen and Kilian, 2006), and *H. parasuis* and *H. paragallinarum* were reclassified as *Glaesserella parasuis* and *Avibacterium paragallinarum*, respectively (Dickerman et al., 2020; Blackall et al., 2005), among other taxonomic reassessments. Despite these ongoing reclassification exercises, several species, such as *H. parainfluenzae, H. ducreyi, H. felis, H. parahaemolyticus, H. paraphrohaemolyticus, H. paracuniculus* and *H. pittmaniae* remain classified within the *Haemophilus* genus, although their exact phylogenetic positions are increasingly under question (Naushad et al., 2015; Christensen et al., 2004, 2014). These discrepancies highlight the need for a comprehensive taxonomic revision of the genus *Haemophilus*, based on genome-scale data, and established within the context of *Pasteurellaceae*. Such a revision would aim to clarify evolutionary relationships, improve classification accuracy and clinical diagnostics to better support research related to these bacteria (Diricks et al., 2022; Harris et al., 2020).

Advances in molecular and genomic approaches have revolutionized bacterial systematics. Techniques, such as 16S rRNA gene sequencing, Multilocus Sequence Analysis (MLSA), and Whole-Genome Sequencing (WGS) have enabled the resolution of phylogenetic relationships with unprecedented detail (Nilsen et al., 2025; Bisgaard and Christensen, 2025; Christensen et al., 2003). Genome-based metrics, such as Average Nucleotide Identity (ANI) and digital DNA–DNA Hybridization (dDDH), provide standardized, quantitative measures for defining bacterial species, exceeding the resolution of traditional phenotypic methods (Meier-Kolthoff et al., 2013; Richter and Rosselló-Móra, 2009). However, frequent HGT events within the family (Redfield et al., 2006) may obscure species boundaries and phylogenetic signals, making genome-wide analyses particularly valuable for distinguishing vertically inherited relationships from horizontally acquired genes (Riesco and Trujillo, 2024; Polz et al., 2013).

In line with these advances, numerous taxonomic revisions have been proposed across the family *Pasteurellaceae* (Christensen et al., 2020a, 2021). Several species formerly assigned to genus *Pasteurella* have been reclassified into other genera, such as *Mannheimia* (Angen et al., 1999) and *Gallibacterium* (Bisgaard et al., 2009). Similarly, phylogenomic analyses have supported the creation of new lineages within *Actinobacillus* (Christensen et al., 2003). These revisions not only expand and clarify existing evolutionary relationships but also improve the accuracy of diagnostic and epidemiological practices.

This study presents comprehensive genomic and phylogenomic analyses, using the type strains representing all validly published genera within *Pasteurellaceae*, with special focus on the historically-diverse and clinically problematic genus, *Haemophilus*. Applying whole-genome sequence data, core-gene phylogeny and genomic similarity metrics (ANI and dDDH), we have reassessed genus boundaries and propose well-supported taxonomic reclassifications. Additionally, we describe a new genus to accommodate a distinct phylogenetic lineage formerly assigned to *Haemophilus*, based on solid genomic, phylogenetic and phenotypic evidence.

Through this integrated approach, we have resolved longstanding taxonomic ambiguities, established a stable genomically informed taxonomy that reflects documented evolutionary relationships and supports improved classification and identification in both clinical and research settings.

## Materials and methods

### Genome data acquisition

Type strain genome sequences for all validly published species within the genus *Haemophilus*, as well as, for all validly published genera within the family *Pasteurellaceae* were retrieved from the National Center for Biotechnology Information (NCBI) GenBank database (Sayers et al., 2025; O’Leary et al., 2024) to build a comprehensive phylogenetic framework. This dataset, including all available genomes from genera closely related to *Haemophilus*, were used to perform a comprehensive genomics-dependent taxonomic reassessment based on proposed minimal standards (Riesco and Trujillo, 2024). All genome data were downloaded on June 4, 2025. Details of all genome sequences analyzed in this study, including strain names, species, accession numbers, genome sizes and source information, are provided in Supplementary Information S1.

### Genome phylogenetic analysis

The genome sequences of the type strains of all species of the genus *Haemophilus* were compared with those of the type strains of species from the other genera within the family *Pasteurellaceae*. Additionally, type strains of species of the most closely-related genera were included to better clarify the classification of *Haemophilus* species. Two core proteomes were calculated, using the M1CR0B1AL1Z3R v2.0 web server (Avram et al., 2019; Shimony et al., 2025), based on the genome sequences of the type strains. Orthologous genes in these genomes were identified, using the same web server^2^. The server extracts orthologous gene sets from each genome and analyzes gene presence-absence patterns. The following parameters were applied: a maximum e-value cutoff of 0.01; percent identity cutoffs of 70 and 40%, both with a coverage cutoff of 70%; and a minimal core genome percentage of 100%, with bootstrap values. Two percent identity thresholds were applied to evaluate how sensitive the core genomes definition was under different levels of stringency. The 70% cutoff provides a conservative estimate of highly conserved orthologs, whereas the 40% cutoff allows the inclusion of more divergent homologous genes. Comparing both approaches enabled assessment of the stability of the core genome and downstream phylogenetic inferences. The concatenated sequences of the resulting core proteomes were used to infer phylogenetic relationships among the strains. Phylogenetic trees were constructed by maximum likelihood (ML). Bootstrap values of 100 replicates were calculated and tree topologies were visualized, using the interactive Tree Of Life iTOL v7.2.2 program, displaying bootstrap values as node labels (Letunic and Bork, 2021). Trees were visualized as unrooted to better assess monophyletic branching patterns. Strains clustering within the same phylogenetic branch were considered to be members of the same phylogenomic-based genus.

### Sequence analysis

The 16S rRNA gene sequences of type strains were retrieved from the NCBI GenBank database (Sayers et al., 2025; O’Leary et al., 2024), based on species listed in the LPSN (Parte, 2014). All sequences were sourced from PCR-amplified DNA and downloaded on June 4, 2025. An individual tree was generated from 100 16S rRNA gene sequences (1,240 nt).

Multiple sequence alignments were conducted, using the hierarchical method in CLUSTAL_W v2.1 (Larkin et al., 2007) and implemented in MEGA11 v11.0.13 (Tamura et al., 2021; Larkin et al., 2007). Automatically-aligned sequences were reviewed and edited manually. Similarities and evolutionary distances were calculated, using Compute Pairwise Distances in MEGA11 (Tamura et al., 2021). Gene distances were derived from nucleotide sequences, using the Jukes-Cantor method (Jukes and Cantor, 1969), and dendrograms were created, using Neighbor-Joining (NJ), Maximum Composite Likelihood and Pairwise deletion methods (Christensen and Olsen, 2023). Bootstrap analysis with 1,000 replicates was performed to assess node support, with bootstrap values greater than 70% indicated at branching points. Tree topologies were visualized with iTOL program v7.2.2, displaying bootstrap values as node labels (Letunic and Bork, 2021). Additionally, evolutionary genetic distances (p-distances) between sequences were calculated in MEGA11. A heatmap showing similarity values (1 − p-distance) was generated in RStudio v.2024.09.0 + 375 (RStudio Team, 2016) and R v4.4.2, using the pheatmap package (Kolde, 2019), applying the Euclidean clustering method.

### Overall genome relatedness indices (OGRI) analysis

Overall Genome Relatedness Indices (OGRI) were calculated, to assess genomic relatedness of both species and genus levels, including ANI, dDDH, Percentage Of Conserved Proteins (POCP), and Average Amino Acid Identity (AAI) (Riesco and Trujillo, 2024). First, pairwise ANI values were estimated, using PyANI v0.2.11 with default parameters (Pritchard et al., 2016) and further confirmed by calculating BLAST-based ANI (ANIb), using JSpeciesWS v5.0.2 (Richter et al., 2016), with ANI values ≥ 95% considered indicative of species-level similarity. Second, dDDH estimates were obtained from the Genome-to-Genome Distance Calculator v3.0 (GGDC; https://ggdc.dsmz.de/, accessed June 2025), using Formula 2 (identities/HSP length), which is recommended for draft genomes, and values ≥70% were used as the species boundary threshold (Meier-Kolthoff et al., 2013). Third, POCP values were determined, following the method of Qin et al. (2014) by performing BLASTp v2.11.0 comparisons of all protein-coding sequences with cutoffs of ≥40% sequence identity and ≥50% alignment coverage (POCP 40–50), implemented through the POCP-nf pipeline (Hölzer, 2024). The resulting values, expressed as the mean proportion of conserved proteins relative to the total number of proteins in each genome pair, were interpreted, using a 50% genus-level threshold. Additionally, a more stringent “70–70 POCP” criterion (≥70% sequence identity and ≥70% alignment coverage) was applied to refine genus delineation. Finally, AAI values were calculated, using CompareM v0.1.2^3^, comparing orthologous protein-coding genes under default parameters and interpreted within the context of proposed genus boundaries (generally 60–80%, although no universal cutoff has been established), in combination with ANI, dDDH, POCP, and phylogenomic analyses (Riesco and Trujillo, 2024; Brenner et al., 2005).

### Analysis of virulence factors

Virulence Factors (VFs) were identified, using the VFanalyzer tool from the Virulence Factor DataBasev2.0 (VFDB; http://www.mgc.ac.cn/VFs/) (Zhou et al., 2025). However, the VFDB VFanalyzer currently lacks specific genus options for several genera within the *Pasteurellaceae* family, including *Pasteurella, Rodentibacter, Aggregatibacter, Actinobacillus*, among others. As a result, the genus *Haemophilus* was chosen as the representative genus for virulence factor detection across the family. This approach allowed for comprehensive screening of known or potential virulence genes and their locations across multiple *Pasteurellaceae* genera despite database limitations, supporting a broad comparative genomic analysis of virulence factor repertoires within the family.

### Functional annotation and phenotype prediction

The bioinformatic tool Protologger v.1.2.0 (Hitch et al., 2021), implemented on the Galaxy platform v22.05 (The Galaxy Community, 2024), was used for phenotype prediction and protologue generation directly from genome sequences. This automated method enables comprehensive functional annotation and taxonomic characterization by analyzing genomic data for phenotypic traits and important taxonomic features.

In parallel, protein-coding sequences (*.faa) were annotated, using a custom pipeline combining Hidden Markov Model-based (HMM-based) searches and pathway completeness analysis. Each file was processed with HMMER v3.3 (hmmscan) (Eddy, 2011) against the KOfam HMM database (hmdb/db_kofam.hmm) (Aramaki et al., 2020) with curated cutoffs (--cut_ga). Tabular outputs were converted, using custom scripts to extract KEGG Orthology (KO) identifiers, generating cleaned KO lists for each genome, as suggested by *give_completeness* tool v1.3.0. Functional completeness of KEGG modules was then assessed, using *give_completeness*, and genome-level completeness tables, merged into a single matrix, with missing modules encoded as zero (GitHub: EBI-Metagenomics/kegg-pathways-completeness-tool).

The matrix was analyzed in RStudio v.2024.09.0 + 375 (R v4.4.2), to examine functional potential across genomes. K-means clustering (10 clusters) grouped modules with similar completeness patterns and KEGG module descriptions were retrieved, via the KEGG REST API, to annotate clusters. Heatmaps were generated with ComplexHeatmap, using a red-to-green gradient to show completeness, with rows split by clusters and word clouds of module descriptions added as row annotations (simplifyEnrichment:anno_word_cloud). Heatmaps were exported as PDF and SVG files, and columns were optionally reordered alphabetically (Sato et al., 2023). This combined approach, using Protologger and HMM/KOfam analysis, offered comprehensive functional annotation and visualization of conserved and variable metabolic capabilities across genomes.

### Cellular fatty acid analysis

All Culture Collection University of Gothenburg (CCUG) strains analyzed in-house were grown on Chocolate Agar (Substrate Department, Sahlgrenska University Hospital, Gothenburg, Sweden) at 37 °C supplemented with 5% CO_2_ for about 18 h. Fresh bacterial biomass (80–100 mg) was then harvested and the extraction of Cellular Fatty Acid-Methyl Esters (CFA-FAMEs) was performed following the protocol described by Sasser (2006). The CFA-FAME profiles of strains were identified, using a HP 5890 Gas Chromatograph (Hewlett-Packard, HP; Palo Alto, United States of America) (Eerola and Lehtonen, 1988) and an in-house variation of the MIDI Sherlock Microbial Identification System (MIDI, Inc., Newark, DE, United States) method^4^.

## Results and discussion

### Phylogenomic analysis

**Figure 1:**
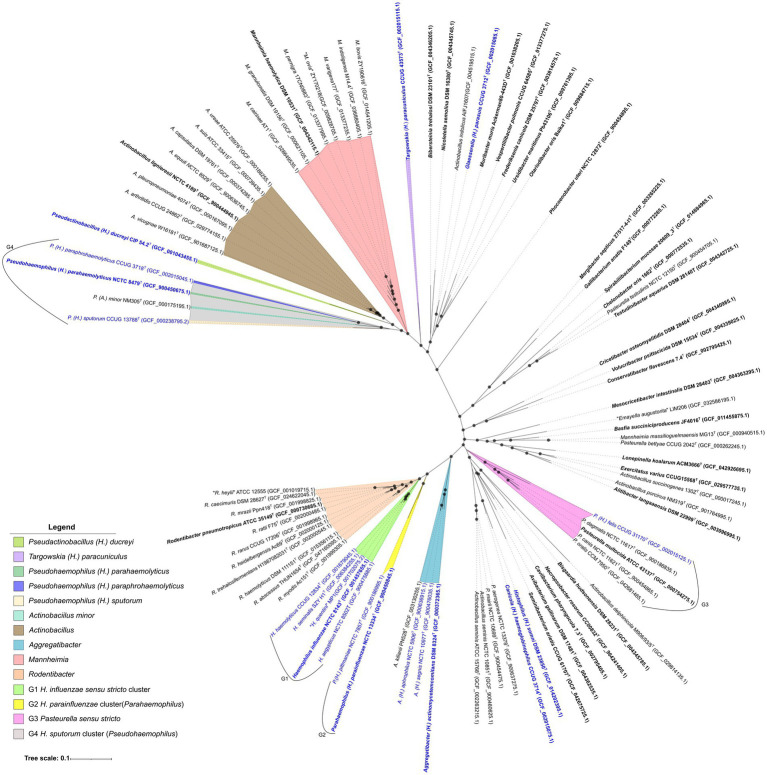
Phylogenetic tree, using M1CR0B1AL1Z3R 2.0 (orthologs criteria: 70% identity; 70% coverage), of the concatenated alignment of 293 monocopy proteins of the core proteome defined in the 90 genomes analyzed within the family *Pasteurellaceae*. The 103,656 amino acid positions were used to construct the tree. Phylogenomic species inside each phylogenetic branch are highlighted with different colors. Type species are labeled in bold text and the type strains of *Haemophilus* species (and synonyms) are highlighted in blue text. Accession numbers of the corresponding genomes are given in brackets. Bootstrap values greater than 80 are indicated in the nodes. Bar, 0.1 substitutions per nucleotide position.

The relationships among the species of the genus *Haemophilus* and of other genera within the family *Pasteurellaceae* were analyzed by determining the core genome. The concatenated core proteomes of the species type strains included 520 proteins at 40% identity and 70% coverage (24.4% of the average proteome size of strains of the family *Pasteurellaceae*) (Supplementary Information S2), while 293 proteins at 70% identity and 70% coverage, representing 13.7% of the average proteome. Importantly, both datasets were used to infer the phylogenomic relationships within the family *Pasteurellaceae* and produced highly similar tree topologies (data not shown), indicating that the phylogenomic relationships are robust to the choice of core genome dataset. For the final phylogenomic analysis, we selected the 70/70% identity-coverage core proteome. The concatenated sequences from this alignment included 103,656 amino acid positions and were used to construct a phylogenomic tree (Figure 1). All branches were strongly supported, showing 100% bootstrap support across 100 replicates.

The phylogenomic tree revealed several well-supported clades within the family *Pasteurellaceae*. The species classified within the genus *Haemophilus* were observed to form six distinct monophyletic groups. Within one of these groups (designated, G1), *H. influenzae sensu stricto* clustered closely with *H. aegyptius, H. haemolyticus, H. seminalis* and *H. quentini*, indicating a recent common ancestor. The *H. parainfluenzae* cluster (designated, G2) and species of the genus *Rodentibacter* clustered separately with both of these groups positioned adjacent to G1 group. *H. parainfluenzae* and *H. pittmaniae* formed a monophyletic cluster, located between the G1 group and the genus *Aggregatibacter,* indicating a distinct evolutionary lineage. *H. felis* clustered within the *Pasteurella sensu stricto* clade (designated, G3), indicating that it is more closely related to species of *Pasteurella* than to those of the genus *Haemophilus*. In contrast, *H. paracuniculus* formed a distinct lineage, compromising a single species, positioned between the species of the genus *Mannheimia* and a larger monophyletic group composed of species of nine different genera: *Bibersteinia trehalosi*; *Nicoletella semolina*; *Glaesserella parasuis*; *Muribacter muris*; *Vespertiliibacter pulmonis*; *Frederiksenia canicola*; *Ursidibacter maritimus* and *Otariodibacter oris*. This isolated phylogenetic position suggests that *H. paracuniculus* represents a unique evolutionary lineage within the family *Pasteurellaceae. H. ducreyi* was observed to form a monophyletic branch with the genus *Actinobacillus*; however, *H. ducreyi* exhibited an earlier divergence, compared with the other *Actinobacillus* species, suggesting greater evolutionary separation. Furthermore, *H. parahaemolyticus, H. paraphrohaemolyticus* and *H. sputorum* clustered with *Actinobacillus minor*, forming a distinct monophyletic lineage (designated, G4), clearly separated from the main *Actinobacillus sensu stricto* clade and from other *Haemophilus* species.

Additionally, the type strains of type species from the genera *Pasteurella, Actinobacillus* and *Mannheimia* were found to cluster within clades comprising species (type strains) from other genera. This suggests potential taxonomic inconsistencies and indicates that some species currently assigned to these genera require reevaluation based on their phylogenomic placement.

### Phylogenetic analysis of 16S rRNA gene sequences

**Figure 2:**
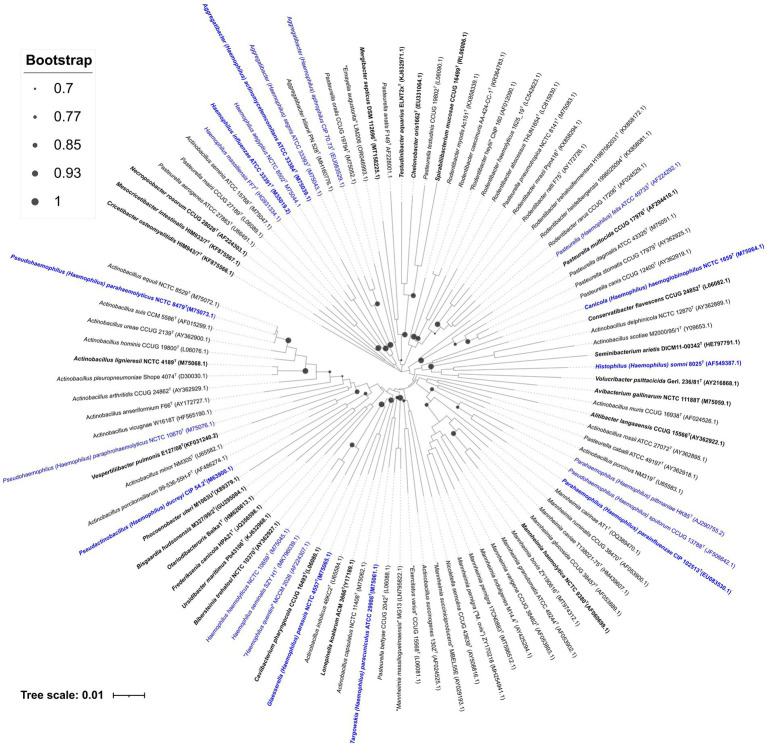
16S rRNA gene-based neighbor-joining tree constructed, using the Jukes–Cantor model, showing the phylogenetic relationships among *Pasteurellaceae* type species, based on 1,240 aligned nucleotide positions. Type strains of the type species are indicated in bold text and the type strains of *Haemophilus* species and synonyms are highlighted in blue text. GenBank accession numbers of the corresponding genes are given in brackets. Bootstrap values (1,000 data resampling) are shown at the nodes. Bar, 0.01 substitutions per nucleotide position.

Phylogenetic analyses were conducted to assess the impact of the 16S rRNA gene, the primary universal phylogenetic marker for identifying new *Pasteurellaceae* (Naushad et al., 2015; Christensen et al., 2003); comparative 16S rRNA gene sequence analyses are fundamental to modern bacterial taxonomy and has been essential for describing new bacterial species (Hackmann, 2025; Johnson et al., 2019). The analyses in this study used archived sequences of 100 16S rRNA genes from the type strains of type species of genera of the *Pasteurellaceae* family, including strains from *Haemophilus, Mannheimia*, *Pasteurella, Rodentibacter, Aggregatibacter, Actinobacillus*, and others (Supplementary Information S1). The sequences were retrieved from the NCBI database, based on species descriptions listed in the List of Prokaryotic Names with Standing in Nomenclature (LPSN) (June 12, 2025). After aligning and trimming the terminal regions, the final alignment contained 1,240 nucleotide positions. A similarity matrix was calculated (Supplementary Information S3), allowing for the determination of clustering values for type strains of species within their respective genera and a Neighbor-Joining phylogenetic tree was generated (Figure 2), with bootstrap support (1,000 replicates), indicating the genetic distances among sequences. The phylogenetic placement of the 100 16S rRNA gene sequences into known genera and species generally agreed with the groupings established from the phylogenomic analysis, with the lowest similarity values among the clustered sequences ranging from 94 to 96%. The *Haemophilus* species analyzed showed distinct clustering patterns in the phylogenetic tree. The G1, *Haemophilus sensu stricto* clade, was divided into two groups: the first included *H. influenzae* and *H. aegyptius*, which could not be clearly distinguished from each other, applying the 98.7% identity threshold (Stackebrandt and Ebers, 2006), leading to their co-clustering; the second group comprised *H. haemolyticus, H. seminalis,* and “*H. quentini*,” which clustered together with species of different genera, including *Glasserella parasuis* and *Caviibacterium pharyngocola*. The G2 group, including *H. pittmaniae* and *H. parainfluenzae*, formed a cluster closer to *Mannheimia*. Additionally, G3 was confirmed to be within the *Pasteurella sensu stricto* group. G4 was divided into two subgroups: *H. paraphrohaemolyticus* and *H. parahaemolyticus*, clustering closely with *Actinobacillus* species; while *H. sputorum* clustered with G2. Finally, *H. ducreyi* grouped near *Phocoenobacter uteri*.

The results of this study highlight the importance of exercising caution when interpreting phylogenetic relationships based on 16S rRNA gene sequences. It is crucial to recognize that taxa, ideally, should form strongly supported monophyletic groups, as the accuracy of phylogenetic inferences depends heavily on the robustness of the clade support. Misinterpreting poorly supported or paraphyletic groupings can lead to incorrect assumptions about evolutionary relationships and taxonomic classifications (Chung et al., 2018). Specifically, the genus *Haemophilus* has traditionally been described as a paraphyletic group, raising questions about its taxonomic boundaries (Christensen et al., 2020a). The findings presented here align with previous research, indicating that species classified as *Haemophilus* actually contain multiple distinct lineages that should be recognized as separate monophyletic groups (Christensen et al., 2007). This evidence not only supports the idea that the genus, as it is currently defined, may include misclassified species but also underscores broader implications of relying solely on 16S rRNA gene-based phylogenies for taxonomic decisions.

### POCP-based delimitation of genera of the family *Pasteurellaceae*

Taxonomic reorganization within the genus *Haemophilus* is necessary due to the existence of several polyphyletic and paraphyletic lineages. Such reorganization requires establishment of clear criteria for defining the acceptable level of diversity within a genus. In this context, genome-based phylogenies should be the primary framework for identifying well-supported monophyletic groups, offering a solid foundation for taxonomic revisions (Rosselló-Mora and Amann, 2001). Within this framework, the usefulness of genome-based similarity indices, such as POCP, as supplementary tools for defining generic boundaries can be evaluated (Qin et al., 2014).

A pairwise POCP matrix was generated for the type strains of 90 species across the genera of the *Pasteurellaceae* family to evaluate genus-level relationships. According to the canonical workflow of Qin et al. (2014), 40–50 POCP (calculated with ≥40% identity and ≥50% coverage), generally can be used to delineate genera, with values greater than 50% indicating that species belong to the same genus. Applying this 50% cutoff threshold would automatically conflict with the requirement of monophyly for taxon delineation. Among the taxa analyzed, only *Actinobacillus delphinicola* fell below this threshold, making it the only distinct genus within the family. All other analyzed species exhibited intra-genus POCP values well above 50%. For example, well-defined *sensu stricto* genera, such as *Rodentibacter* and *Mannheimia*, shared more than 80% POCP among their constituent species and exhibited values ranging from 60 to 80%, when compared with strains from closely related genera. Similarly, *Actinobacillus sensu stricto* group exhibited the same pattern; although, some *Actinobacillus* species appeared to cross over into other generic groups. As a result, the 50% POCP boundary is not an appropriate metric for delineating genera within *Pasteurellaceae*, leading instead to of multiple genera into broad clusters of overlapping groups. However, applying a stricter threshold of 80% does not necessarily improve the resolution of genus-level clustering (Supplementary Information S4).

The analysis was subsequently modified to use 70–70 POCP (calculated with ≥70% identity and ≥70% coverage), to achieve higher resolution. In Supplementary Information S5, the results of this modified POCP was applied. Considering the 40% threshold for the resulting values, three well-defined and clearly separated clusters emerged, while four type species (*Actinobacillus delphinicola, Gallibacterium anatis,* and *Mergibacter septicus*) remained isolated. Within the two main clusters, the *Haemophilus* species were split. One cluster contained *H. influenzae sensu stricto* (G1) and the *H. parainfluenzae* group (G2), together with species of other genera, while the other cluster included *H. ducreyi* and the G3 group with additional species of other genera. Among these other genera, such as *Actinobacillus, Aggregatibacter, Mannheimia, Pasteurella* and *Rodentibacter*, their species distribution matched their respective phylogenomic clusters. These results suggest that *Haemophilus* should be split into multiple distinct genera, whereas the other genera remain largely cohesive as defined clusters. However, this criterion, alone, is not enough to fully distinguish all currently known genera. In such a heterogeneous family, a more stringent, 70–70 POCP analysis highlights the potential for identifying inconsistencies in taxonomy at both the family and genus levels. Furthermore, applying a stricter threshold (≥70%) enabled clear differentiation of all defined and validated genera within *Pasteurellaceae*. The resulting POCP relationships were largely congruent with the core-genome phylogeny, with each genus forming distinct, single-lineage branches (Figure 1). Minor topological differences were limited to closely related taxa, while the overall clustering pattern closely matched the core-genome tree.

The use of 40–50 POCP has been shown to be ineffective in delineating genera within the families *Bacillaceae* (Aliyu et al., 2016), *Burkholderiaceae* (Lopes-Santos et al., 2017), *Neisseriaceae* (Li et al., 2017), *Rhodobacteraceae* (Wirth and Whitman, 2018) and *Methylococcaceae* (Orata et al., 2018), among others. While these findings collectively demonstrate both the utility and limitations of POCP for genus circumscription within *Pasteurellaceae*, they also highlight its constraints. Conventional POCP thresholds do not perform well in families characterized by high rates of horizontal gene transfer, as genomic mosaicism obscures clear genus boundaries (Polz et al., 2013; Riesco and Trujillo, 2024). Although more stringent thresholds (≥70% identity and coverage) improved congruence with genome-based phylogenies, partly by reducing the impact of horizontally acquired genes, 70–70 POCP values alone were insufficient to fully resolve genus-level diversity and evolutionary relationships. Additional genomic and phenotypic analyses are needed to ensure robust taxonomic delineation.

### Average amino acid identity

Average Amino acid Identity (AAI), together with POCP, is one of the most frequently used genome-based similarity indices for genus delineation (Barco et al., 2020). Although AAI thresholds for defining genera are generally suggested to fall within the range of 60–80% (Liao et al., 2020; Rodriguez-R and Konstantinidis, 2014; Konstantinidis and Tiedje, 2005), in practice, many taxa do not consistently meet these criteria (Riesco and Trujillo, 2024). This raises the question of whether standard AAI cutoffs are always suitable, particularly regarding maintaining genus-level monophyly (Orata et al., 2018). This is because taxonomic ranks have been applied unevenly across the tree of life, with highly studied groups tending to be split into greater numbers of taxa than the less studied groups (Parks et al., 2018).

In our analysis, pairwise AAI values were calculated for the type strains of 90 type species of genera of the *Pasteurellaceae* family, to assess genus-level boundaries. The AAI values obtained ranged from 63.9 to 100% (Supplementary Information S6). Species clustering within the same genus exhibited AAI values of ≥80%, indicating substantial proteomic similarities within these groups. In contrast, most inter-generic comparisons yielded AAI values below this threshold, although the majority were still ≥60%. Using a 60% threshold for genus delineation would group together the distinct genera of the family, as happens in *Enterobacteriaceae* and other well-studied groups (Parks et al., 2018; Luo et al., 2014), resulting in paraphyly or polyphyly, which should be avoided. In contrast, applying ≥ 80% AAI as the lower limit for genus delineation resulted in between 33 and 35 inferred monophyletic genera, which largely preserved the current classification of the analyzed genomes and further supported the genus boundaries identified through 70–70 POCP and core genome phylogeny (Figure 1).

Representative cases illustrate both congruence and conflict between AAI values and expected genus boundaries. For example, *H. ducreyi* clustered with *Actinobacillus sensu stricto*, exhibiting AAI values greater than 80%. Deviations from this pattern were also observed. *H. parainfluenzae* group (G2), displayed AAI values exceeding 82%, with both the *H. influenzae sensu stricto* cluster (G1), and members of the *Rodentibacter* genus. Likewise, *H. felis* did not group within the *Pasteurella sensu stricto*, instead showing intermediate AAI values (77.9–78.5%). In addition, *H. sputorum*, *H. paraphrohaemolyticus*, *H. parahaemolyticus*, and *Actinobacillus minor* distinct cluster (G4), although the AAI value between *H. sputorum* and *A. minor* was observed to be 79.3%. Notably, *Actinobacillus minor* exhibited AAI values above 80% with four type species of *Actinobacillus sensu stricto*, further blurring the boundaries between these taxa.

These cases demonstrate that AAI, while generally dependable, may not always accurately define genus boundaries when used alone. This shortcoming is especially clear in groups where high proteomic similarities exists across different evolutionary lineages, which can result from shared ancestry, convergent functional constraints or the widespread effect of horizontal gene transfer (Polz et al., 2013). In such instances, AAI can mask true evolutionary relationships by overestimating the similarities between unrelated groups (Gerhardt et al., 2025; Orata et al., 2018). Additionally, AAI values can vary, as different prokaryotic taxa, even those closely related, may evolve at different rates, depending on their ecological niches, selective pressures and life history traits (Ramette and Tiedje, 2007). Moreover, the absence of a universally accepted AAI threshold for genus delimitation, mainly caused by the uneven application of taxonomic ranks across the tree of life, and reflected by the proposed cutoffs, ranging broadly from 60 to 80% (Parks et al., 2018; Luo et al., 2014), further complicates its use.

Overall, these findings show that, while AAI is a useful supplementary tool for genus delimitation in *Pasteurellaceae,* it should not be used as the only criterion. Instead, genus assignments should combine AAI with POCP, core genome phylogeny and other genomic metrics to make sure that the taxonomic demarcations are robust and phylogenetically consistent.

### Average nucleotide identity and digital DNA–DNA hybridization

Although genus-level analyses (core genome phylogeny, POCP and AAI) significantly enhanced the resolution of *Pasteurellaceae* taxonomy and resolved most cases of polyphyly and paraphyly within *Haemophilus*, issues of species-level misclassification still persist. To assess genomic similarities at finer taxonomic levels, ANI and dDDH values were calculated for the type strains of 90 type species of genera across the *Pasteurellaceae* family.

ANI values were consistently high, generally exceeding 79–82%, as shown in Supplementary Informations S7, S8, within groups defined by the 70–70 POCP criterion. These values distinguish groupings that correspond to known or potential genera. This aligns with the intragenic values of 77–92% proposed by Rosselló-Móra and Amann (2015) and the results suggest that this range could serve for effective genus delineation within the family *Pasteurellaceae*, further corroborating the clusters identified through POCP and core-genome phylogeny. As a supplementary measure to ANI, dDDH values were also analyzed, revealing a similar pattern at the species level (Supplementary Information S9). dDDH values above 70% were observed only between some strains of different species, such as *H. influenzae* and *H. aegyptius*, while nearly all other pairwise comparisons, including those within the same genus, were below 59%. This confirms dDDH to be reliable for species delineation but offers limited reliability for genus-level classification within *Pasteurellaceae* (Christensen et al., 2020a).

Overall, ANI and dDDH offer additional support for the genus clusters identified through POCP and core genome phylogeny, while also highlighting taxa with intermediate or ambiguous placements. These results underscore the importance of an integrated approach to *Pasteurellaceae* taxonomy, combining multiple genomic metrics (ANI, dDDH, POCP and AAI) with phylogenomic analyses. Such an approach allows for robust, phylogenetically consistent, and biologically meaningful genus definitions and helps identify transitional, misclassified or evolutionarily atypical taxa, as shown by *H. parahaemolyticus*.

### Virulence factor profiles across *Pasteurellaceae*

Comparative genomic analysis of species of genera within *Pasteurellaceae* identified both conserved and genus-specific patterns in virulence factor (VF) distribution, reflecting the existing host adaptation and ecological niche specialization within the family (Abdel-Glil et al., 2023; De Luca et al., 2021; Ramette and Tiedje, 2007; Gioia et al., 2006). A total of 239 virulence-associated genes were found across the genome sequences of the type strains of 90 species examined (Supplementary Information S10). On average, each strain contained approximately 29.9% of the total VF repertoire (~81 VF). However, substantial variation was observed, considering the strain with the highest number of virulence factors as the reference, the average VF content reached as high as 79.3% of the total VF set. Notably, *H. sputorum* and *A. minor* lacked detectable VF genes. Excluding these strains, the minimum relative VF content per genome increased to 61.1%, indicating that most species of *Pasteurellaceae* genera encode a significant array of virulence factors.

Analyzing VF distribution provides additional resolution for species and genus delineation, as variations in the presence or absence of specific virulence genes can reflect evolutionary divergence and ecological specialization among closely related strains. Among the VFs analyzed, 48 factors were found in more than 80% of strains, with 30 of these detected in more than 95% of the genomes, forming a conserved ‘soft-core’ set of virulence determinants within the family (Supplementary Information S10). These highly conserved factors include components of type IV pili (*comE*/*pilQ*, *pilB*), which are essential for bacterial attachment (McCallum et al., 2019; Melville and Craig, 2013), and lipooligosaccharide (LOS) biosynthesis genes, such as *gmhA/lpcA*, *htrB*, *kdsA*, *kdsB*, *kdtA*, *kpsF*, and the *lpx* gene cluster (*lpxA*, *lpxB*, *lpxC*, *lpxD*, *lpxH*, *lpxK*), which are crucial for immune modulation (Tzeng et al., 2024; Plant et al., 2006; Kahler and Stephens, 1998). Genes involved in exopolysaccharide synthesis (*galE*, *galU*, *manB*, *mrsA*/*glmM*, *pgi*) and heme biosynthesis (*hemH*, *hemN*, *hemY*), which are important for iron acquisition (Layer, 2021), were also present across all strains of all species analyzed, highlighting their vital roles in *Pasteurellaceae* virulence.

Within the genus *Haemophilus* and closely related genera, several genus- and species-specific virulence factors are discernible, once the conserved VFs within the *Pasteurellaceae* family are excluded. *H. ducreyi*, notably, shares several key virulence genes with species of *Actinobacillus sensu stricto*, including *hmw1C*, *pilA* and *pilC*, which are linked to pilus assembly and adherence. Additionally, genes involved in LOS biosynthesis and modification, such as *kdkA*, *lsgD*, *lsgE*, *lsgF*, *rffG*, *waaQ* and *wecA*, are conserved between these species, indicating shared mechanisms of immune evasion and serum resistance. Other shared genes include *manA* and *ompP2*, as well as heme biosynthesis genes, *hemD*, *hemM* and *hemX*. The superoxide dismutase gene, *sodCI*, which plays a role in oxidative stress resistance (Lv et al., 2025), is also conserved.

Similarly, the *H. influenzae sensu stricto* (G1) and *H. parainfluenzae* cluster (G2) share virulence factors, including *oapA, iga1, lsgD*, *lsgE*, *lsgF*, *waaQ*, *wecA*, *ompP2*, *hemM* and *hemX*. Notable species-specific differences include the presence of *ppkA* in 100% of G2 but its absence in G1. Conversely, G1 uniquely harbors *lsgA*, *lsgB*, *lsgC*, *neuA* and *yhbX*, which are absent in G2. These distinctions reflect species-specific adaptations that influence virulence and host interaction. *H. felis* and species of *Pasteurella sensu stricto* show overlap in virulence genes, such as *ompP5*, *pilA* and *pilC*, involved in pilus assembly and adherence, both also share LOS biosynthesis and modification genes, including *lsgA*, *lsgD*, *lsgE*, *neuA*, *rffG*, *wecA* and *manA*. Iron acquisition systems are represented by *hitA, hitB* and *hitC*, as well as an extensive set of heme biosynthesis genes (*hemA*, *hemC*, *hemD*, *hemE*, *hemG*, *hemL* and *hemX*). The catalase gene *katA*, linked to oxidative stress defense, was also conserved among G3 but absent in G1, G2 and G4. These shared virulence determinants suggest common pathogenic strategies within these genera.

In summary, while the species of the genera of *Pasteurellaceae* share a conserved core of virulence genes that are essential for colonization, immune evasion and iron acquisition, genus- and species-specific variations highlight evolutionary divergence and niche specialization. This intricate balance of conserved and unique virulence factors underpins the diverse pathogenic potential observed across the family.

### Functional annotation and phenotypic characterization

Phenotypic data from experimental assays found in the literature were combined with functional and metabolic annotations from genomic analyses, to characterize the different genera within the family *Pasteurellaceae*. Protein-coding sequences predicted from the genomes were re-annotated, using Protologger and InterProScan, offering complementary insights into functional capacity and metabolism.

#### Phenotypic data from the literature

Phenotypic traits compiled from multiple sources encompassed metabolic capacities, substrate utilization patterns and additional physiological characteristics (Supplementary Information S11). These data were used to compare and describe species and genera across the *Pasteurellaceae* family, revealing conserved and variable features. Clear phenotypic patterns emerged that paralleled the genomic groupings G1–G4. The three predominant CFAs in most *Pasteurellaceae* strains were myristic acid (14–30%), palmitic acid (19–36%), and the monounsaturated fatty acid C16:1 ω7c (23–39%; Equivalent Chain Length (ECL) 15.819) (Supplementary Information S11).

Within G1, the different species showed nearly indistinguishable biochemical profiles, including shared V-factor dependence, absence of porphyrin synthesis, and consistent catalase and oxidase positivity. All members were also urease-positive and able to utilize D-galactose. These phenotypic similarities are consistent with their tight co-clustering in the genomic analyses. Their major fatty acids were palmitic acid (36%), C16:1 ω7c (30%) and myristic acid (19%), followed by a cellular fatty acid with ECL 15.485 (9%, 14,0 3OH and/or 16,1 ISO I = Summed Feature 2), and similar amounts (2%) of stearic acid, C18:1 ω9c and a fatty acid with ECL 17.724 (18,2 ω6,9c and/ or 18,0 ANTE = Summed Feature 5).

The G2 group was characterized phenotypically by V-factor independence and positive porphyrin synthesis. Hemolysis was variable in *H. parainfluenzae* but consistently positive in *H. pittmaniae*. In terms of carbohydrate utilization, the group was positive for D-mannose, D-fructose and D-galactose. Their fatty acid profiles were 16:1 ω7c (37%), palmitic acid (30%) and myristic acid (19%), followed by small proportions of a CFA with ECL 15.485 (7%, Summed Feature 2), C18:1 ω9c (1%), as well as trace amounts of stearic acid.

Phenotypic traits within G3 included catalase activity and uniformly positive P-nitrophenyl α-D-glucopyranoside (PNPG) activity, as well as positive reactions for D-galactose and D-mannitol utilization. These characteristics were consistent with *Pasteurella sensu stricto* species identities, which typically exhibits positive oxidase activity. In contrast, *H. felis* differed from classical *Pasteurella* species by showing a negative oxidase reaction and requiring V-factor for growth The main fatty acids were observed to be palmitic acid (29%), a CFA with ECL 17.724 (17%) and C16:1 ω7c (13%), followed by lauric acid (10%), stearic acid (8%), myristic acid (7%), a fatty acid with ECL 15.485 (7%, Summed Feature 2) and C18:1 ω9c (6%). However, *H. felis* displayed considerable differences with *Pasteurella sensu stricto* CFA profiles, with outstanding deviations of lauric acid, myristic acid, C16:1 ω7c fatty acid profiles. *H. felis* exhibited higher levels of lauric acid (10%) than strains of *Pasteurella sensu stricto* species (non-detected to 1%), lower amounts of myristic acid (7%) than in *Pasteurella sensu stricto* species (18–27%) and slightly lower amounts of C16:1 ω7c (13%), when compared with *Pasteurella sensu stricto* species, 23–35% (Supplementary Information S11).

G4 was characterized phenotypically by positive oxidase, V-factor dependence, positive porphyrin synthesis, *β*-hemolysis, positive urease, and positive reactions for D-fructose and β-galactosidase. *A. minor*, for which fewer phenotypic tests have been recorded, differed by its lack of β-hemolysis and variations in several carbohydrate utilization tests. The species fatty acid profiles (when available) were characterized by high amounts of myristic acid (23–33%), C16:1 ω7c (32–35%) and palmitic acid (16–31%), while lower amounts of CFA Summed feature 2 (7–9%), Summed feature 5 (2–4%) and minor amounts of C18:1 ω9c (1–2%) were noted. Notwithstanding, only 2 of the 4 species included in G4 were compared, as fatty acid profiles of *H. sputorum* and *A. minor* were not available and, therefore, only *H. parahaemolyticus* and *H. paraphrohaemolyticus* were compared (Supplementary Information S11).

Finally, *H. ducreyi* exhibited a markedly reduced phenotypic profile, including positive oxidase activity, negative catalase activity and low variability of carbohydrate use. The fatty acid profiles exhibited fairly similar amounts of palmitic acid (24%), monounsaturated C16:1 ω7c (23%) and myristic acid (19%), followed by a fatty acid with ECL 17.724 (11%, Summed feature 5), monounsaturated C18:1 ω9c (10%, ECL 17.769), a fatty acid with ECL 15.485 (6%, Summed Feature 2), as well as stearic acid (5%) and minor amounts of a fatty acid with ECL 10.915 (2%, C12,0 ALDE) (Supplementary Information S11). Overall, phenotypic profiles across G1–G4 largely mirrored the evident phylogenetic reconstruction as elucidated by genomic analysis. However, incomplete phenotypic data for several taxa prevented direct comparisons among some species historically suspected of misclassification. This underscores a persistent challenge in *Pasteurellaceae* taxonomy: gaps in phenotypic characterization continue to limit accurate assessments of species relationships and boundaries.

#### Functional annotation with Protologger

Functional annotation, using Protologger genome-based analysis, provided a comprehensive overview of protein sequences based on homology and domain architecture, revealing functional categories beyond traditional virulence factors and highlighting the broader metabolic and phenotypic potential of the analyzed strains (Supplementary Information S12). Across the type strains of 90 species of genera of the family *Pasteurellaceae*, Protologger detected an average of 220 transporters, 36 secretion-related genes, and 741 unique enzymes. Additionally, it predicted the carbon sources utilized by each strain, identified antibiotic resistance determinants, characterized carbohydrate-active enzymes (CAZymes) and annotated glycosyltransferase families, among other functional features.

Focusing on the differentiation of the *Haemophilus* complex, Protologger predicted that siroheme biosynthesis from glutamate is present in the species of G2, *H. parainfluenzae*, and *H. pittmaniae*, but absent in other *Haemophilus* species. Analysis of carbon source utilization revealed that species of G4, *H. sputorum, A. minor, H. parahaemolyticus, H. paraphrohaemolyticus*, and G2, *H. parainfluenzae*, and *H. pittmaniae*, are predicted to metabolize both sucrose and starch. In contrast, G1 species, including *H. influenzae, H. aegyptius* and *H. haemolyticus*, appear limited to starch utilization, while, *H. seminalis* (also G1) is predicted to utilize only sucrose.

Unique metabolic traits were also observed among specific groups at the genomic level. Members of G1, the *Haemophilus sensu stricto*, were predicted to degrade ribose, a capability not detected in other *Haemophilus* species. Within G2, the *H. parainfluenzae* group, all species were predicted to degrade lactose and possess the glycoside hydrolase family GH2; G4, the *H. sputorum* group, also metabolizes lactose, except for *H. parahaemolyticus.* Furthermore, all members of this group carried genes for lactaldehyde degradation, a trait absent in other *Haemophilus* species. As for *H. ducreyi*, no distinct functional features differentiating it from other species could be identified.

Overall, Protologger offered detailed insights into the metabolic diversity and potential phenotypic differences within the *Haemophilus* complex, emphasizing group- and species-specific capabilities that may explain ecological and pathogenic differences.

#### Metabolic pathway analysis

**Figure 3:**
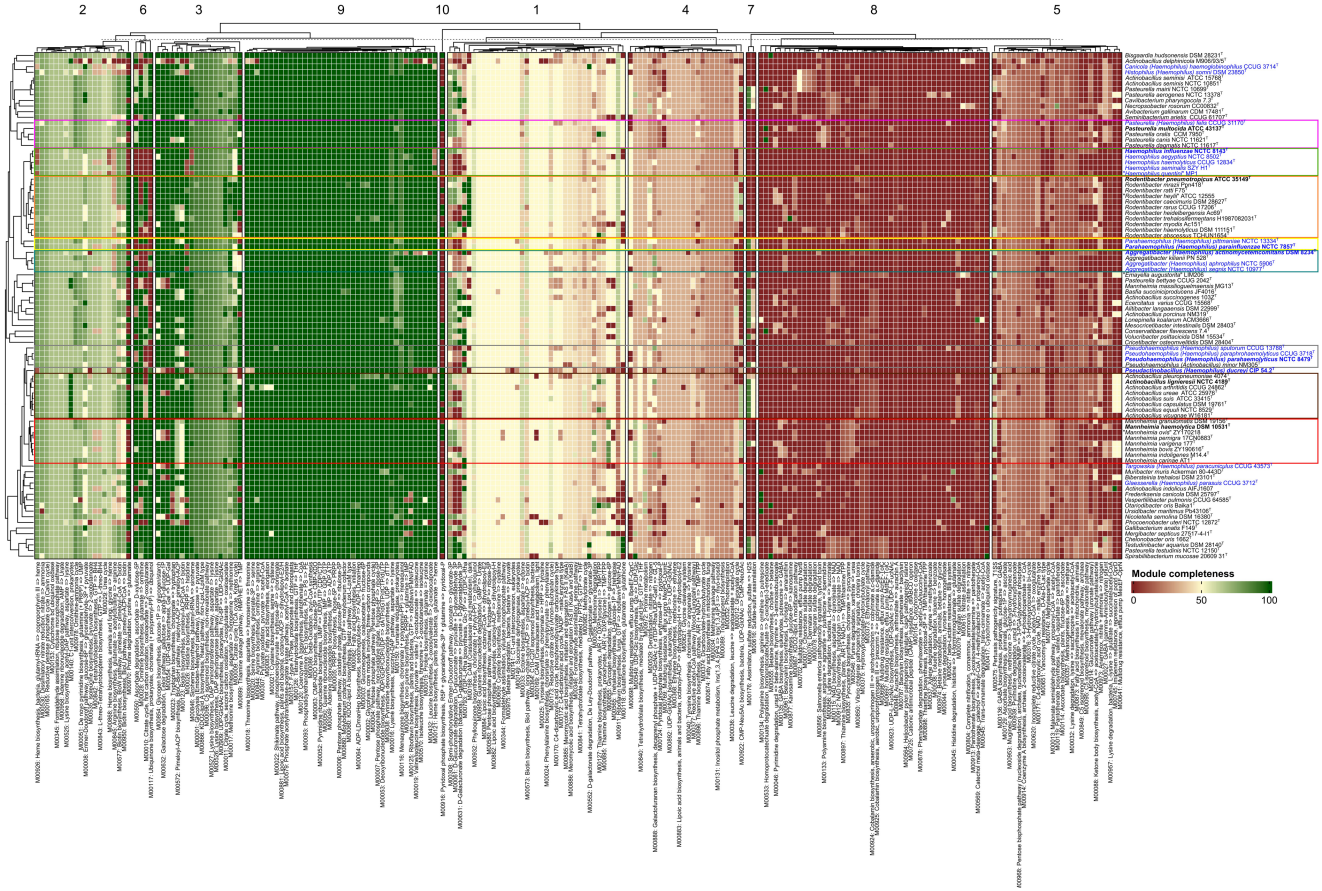
Heatmap of module completeness among *Pasteurellaceae* (90 genomes) based on type species of genera closely related to the genus *Haemophilus* are highlighted in bold. Type strains of the type species are labeled in bold, and *Haemophilus* type species and synonyms are highlighted in blue. Different colors indicate the closely related *Haemophilus*-associated genera: G1, green; G2, yellow; G3, pink; G4, gray; *Rodentibacter*, orange; *Aggregatibacter*, blue; *Actinobacillus*.

The presence and completeness of metabolic pathways across the genomes was determined, using InterProScan. Completeness values were summarized in a heatmap (Figure 3 and Supplementary Information S13), illustrating conserved modules throughout the family and others with variable representation. Based on K-means clustering of KEGG module completeness profiles across genomes, 10 clusters of modules were identified. These clusters represent groups of metabolic pathways sharing similar completeness patterns among the analyzed taxa. Clusters 3 and 9 contained the most complete pathway modules, while clusters 5, 7 and 8 showed the highest number of unique features, being largely absent in the majority of the 90 strains analyzed.

Overall, 36 modules were nearly complete (>95%) in most analyzed genomes. These modules covered key functional categories, including amino acid metabolism, carbohydrate metabolism, energy metabolism, glycan metabolism, lipid metabolism, nucleotide metabolism and cofactor/vitamin metabolism. Within these categories, modules related to central carbohydrate pathways (glycolysis, gluconeogenesis, pentose phosphate pathway), amino acid biosynthesis (proline, lysine, threonine, branched-chain amino acids, cysteine, shikimate pathway), nucleotide biosynthesis (purines and pyrimidines), lipid/fatty acid metabolism and several cofactor/vitamin biosynthesis pathways (riboflavin, heme, tetrahydrofolate, menaquinone) were widely represented. Of these, 14 modules were universally conserved (100% completeness). These included, PRPP biosynthesis, the oxidative phase of the pentose phosphate pathway, adenine ribonucleotide biosynthesis, guanine ribonucleotide biosynthesis, pyrimidine ribonucleotide biosynthesis, CMP-KDO biosynthesis, β-oxidation (acyl-CoA synthesis), phosphatidyl-ethanolamine biosynthesis, coenzyme A biosynthesis, tetrahydrofolate biosynthesis, F-type ATPase, the pathway of phosphate acetyltransferase–acetate kinase and lipoic acid biosynthesis.

These modules represent essential processes necessary for cell survival and the uniformity of these modules across all strains emphasizes their roles as metabolic standards of the family. In contrast, the other clustered groups of modules exhibited varying levels of completeness, which may indicate genus- or species-specific ecological and host-related adaptations (Supplementary Information S13).

Focusing on the *Haemophilus* groups defined in this study and their closely related genera, the analysis revealed several distinctive patterns besides the conserved core modules. A few examples of these unique traits are outlined below. Assimilatory sulfate reduction was identified in the G2, *H. parainfluenzae* group (and in *H. sputorum* only), providing a clear metabolic feature that distinguishes this group from other *Haemophilus* lineages.

Heme and siroheme biosynthesis pathways were found in the G2 *H. parainfluenzae* group and were detected only in *H. seminalis* within G1. In contrast, ascorbate and ubiquinone biosynthesis pathways were absent in G2 and G1 but were present in G4 and *H. ducreyi.*

Pyridoxal-P biosynthesis was limited to *H. influenzae, H. aegyptius* and *H. felis*, while ubiquinone biosynthesis was observed to be unique to *H. felis*. Notably, *H. ducreyi* lacked several amino acid biosynthesis pathways, including valine/isoleucine biosynthesis, isoleucine biosynthesis, proline biosynthesis, leucine biosynthesis and heme biosynthesis, suggesting a reduced metabolic range, compared with other members of the genus. Additionally, proline degradation pathways were observed in *H. sputorum, Aggregatibacter minor, H. parahemolyticus* and *H. pittmaniae*, and they were consistently conserved across the genus *Rodentibacter*, further emphasizing the variability in metabolic specialization among different groups.

Taken together, these examples demonstrate the type of group-specific patterns detected, many of which may serve as useful metabolic markers for distinguishing lineages, including those within the *Haemophilus* complex. However, the diversity of complete pathways across genomic groups also highlights the significant divergence within *Pasteurellaceae*, emphasizing the difficulty of defining clear genus-level traits based solely on metabolic features derived only from type strains. Thus, within this family, metabolic pathway analysis is likely to be most informative at the species level within established genera, rather than for defining genus-level boundaries. Nevertheless, a thorough analysis of each species and its strains would be necessary to confirm this. Additionally, more than 56 pathways were identified at intermediate completeness (40–70%), further highlighting the variability across taxa and the presence of lineage-specific modules. Some of these partially represented pathways may also reflect uneven exploration of different genera and species, with well-studied pathogens being better characterized than less-studied lineages. These partially represented pathways could result from several factors: incomplete annotation due to yet undiscovered or poorly characterized genes; genuine absence of certain functions in specific groups divergence leading to alternative metabolic routes and limitations related to genome quality (e.g., fragmented assemblies causing missing or truncated genes).

### Summary of key findings

**Figure 4:**
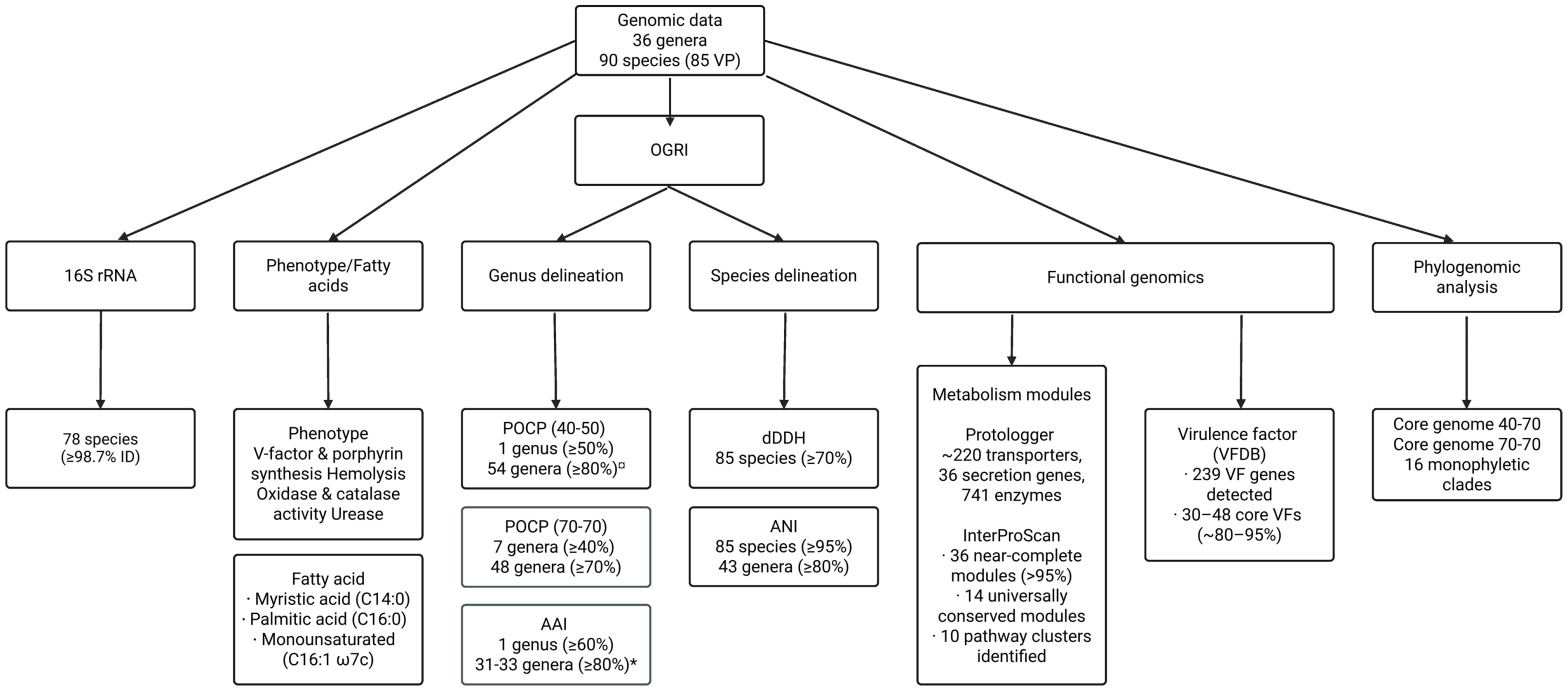
Workflow summarizing the genomic, phylogenomic, and phenotypic approaches used to delineate species of genera and genera within *Pasteurellaceae*. Genomic data were analyzed through Overall Genome Relatedness Indices (OGRI), including POCP and AAI for genus delineation and dDDH and ANI for species delineation. Thresholds and outcomes for each method are indicated within the corresponding boxes. ^¤^Different threshold was applied because 70% POCP failed to cluster genera. ^*^Clustering outcomes depended on whether certain exceptions were considered.

In summary, combining different genomic methods, such as core genome phylogeny, 16S rRNA gene sequence analysis, POCP, AAI, ANI, dDDH, virulence factor profiling and functional profile annotation, creates a strong framework for reevaluating the taxonomy of the species of *Haemophilus* and the family *Pasteurellaceae* (Figure 4). Phylogenomic analyses confirmed that the genus *Haemophilus*, as currently recognized, is in fact, polyphyletic, consisting of multiple well-supported groups (Christensen and Bisgaard, 2018; Naushad et al., 2015). Notably, *H. parahaemolyticus*, *H. paraphrohaemolyticus* and *H. sputorum* cluster monophyletically with *Actinobacillus minor*, supporting their reclassifications into a new genus. Similarly, *H. ducreyi* shows closer relationships with species of *Actinobacillus* yet remains outside the *Actinobacillus sensu stricto* group as a distinct single-species lineage, similar to *H. paracuniculus* with other genera of *Pasteurellaceae*. Each lineage is currently represented by a single species. Establishing new monotypic genera for these taxa would serve as references or anchor points for future sampling that may reveal additional related species. Moreover, *H. felis* clusters with species of the genus *Pasteurella*, revealing issues with current classifications.

Genomic similarity metrics reinforced these findings. ANI and dDDH reliably define species boundaries but offer limited resolution at higher taxonomic levels. In contrast, POCP and AAI demonstrated that conventional genus-level thresholds (POCP ~40–50% and AAI ~ 60–80%) are insufficient for lineages with indications of extensive horizontal gene transfer (Riesco and Trujillo, 2024; Orata et al., 2018; Wirth and Whitman, 2018; Li et al., 2017; Lopes-Santos et al., 2017; Aliyu et al., 2016; Rodriguez-R and Konstantinidis, 2014), while more stringent cutoffs (≥70%) improved alignment with phylogenomic data. We therefore adopted more stringent genus-level cut-offs: POCP ≥70% together with AAI > 80%. These values reproduced better the phylogenomic structure of *Haemophilus* and allied *Pasteurellaceae* lineages. They also reduced the risk of merging ecologically and evolutionarily coherent clades into artificially broad genera. In this context, the ≥70% thresholds function as conservative genus-level criteria tailored to the evolutionary dynamics of these taxa, improving congruence between genome similarities and tree-based relationships and making the resulting genus assignments more transparent and reproducible.

Comparative analysis of virulence factors uncovered a conserved core set of genes necessary for colonization, iron acquisition and immune evasion, along with genus- and species-specific determinants that indicate evolutionary divergence and host adaptation. These functional insights provide further biological support for the proposed taxonomic revisions. However, these virulence profiles should be interpreted with caution, as VFDB coverage remains incomplete and annotations rely largely on generalized *Haemophilus* reference profiles, which may not fully capture lineage-specific determinants across the newly proposed genera.

Meanwhile, the metabolic pathway analysis, based on InterProScan, revealed both conserved and variable modules across *Pasteurellaceae*. Of the 36 nearly complete modules, 14 were universally conserved, representing key processes, such as central carbon metabolism, nucleotide and lipid biosynthesis, cofactor production and energy generation. Beyond these core modules, many pathways showed intermediate levels of completeness (40–70%) or lineage-specific patterns, especially within *Haemophilus* groups. Traits, such as assimilatory sulfate reduction in *H. parainfluenzae*, pyridoxal-P biosynthesis in *H. influenzae* and the lack of several amino acid pathways in *H. ducreyi*, demonstrate how integrated metabolic profiling can identify functional differences that are not visible from genomic data alone. These results highlight potential problems in relying only on genomic or phenotypic data, which can be misleading (Jiao et al., 2024; Chan et al., 2012; Maughan and Van der Auwera, 2011); however, combining both methods provides clearer differentiation of lineages within a taxonomically complex family such as *Pasteurellaceae*.

Despite the robustness of the integrative genomic framework presented here, several limitations should be acknowledged. The analyses are based on type strains and broader intraspecific sampling may reveal additional diversity that could further refine genus-level boundaries. Moreover, phenotypic and ecological data remain incomplete for some taxa and genome-based similarity metrics, although powerful, cannot fully substitute for comprehensive physiological characterization. Future studies incorporating expanded strain collections, detailed phenotypic analyses and improved functional databases will help refine and validate the proposed taxonomic framework.

## Conclusion

Overall, this study offers a comprehensive and solid basis for refining the taxonomic boundaries of the family *Pasteurellaceae*. This integrated approach (i) addresses the long-standing polyphyly of *Haemophilus*, (ii) detects misclassified species, (iii) supports the recognition of new genera, including reclassification of *H. parahaemolyticus* and related taxa, and (iv) emphasizes lineage-specific metabolic and functional traits that improve our understanding of evolutionary divergence.

In conclusion, current classifications among genera of *Pasteurellaceae* need substantial revision. Future taxonomic approaches should go beyond using only a single marker or data type and, instead, adopt integrative strategies that combine phylogenomics, multi-metric genomic analyses and phenotypic and metabolic information. Such an approach offers a consistent, robust and biologically meaningful taxonomy and helps identify functionally and evolutionarily distinct lineages within complex bacterial families. Phylogenomic and comparative genomic analyses consistently resolve five well-supported clades within the current assemblage of *Haemophilus* species. These clades correspond to evolutionarily and genomically coherent lineages that differ from *Haemophilus sensu stricto* and other genera of *Pasteurellaceae* by multiple independent criteria. Accordingly, we propose the recognition of four novel genera. *Parahaemophilus* gen. nov., (G2), accommodates *H. parainfluenzae* and *H. pittmaniae. Pseudohaemophilus* gen. nov., (G4), encompasses *H. parahaemolyticus*, *H. paraphrohaemolyticus*, *H. sputorum* and *A. minor*. The single-species lineages represented by *H. ducreyi* and *H. paracuniculus* are phylogenetically distinct, as well, and are proposed here as members of new genera (*Pseudactinobacillus* gen. nov. and *Targowskia* gen. nov., respectively). *H. felis* is reassigned as a new combination within *Pasteurella sensu stricto* (G3). All of these proposed changes based on data of the type strains of the type species are made to be able to determine newly recognized phylogenetic relationships while establishing pragmatic anchor points for nomenclatural stability. This study may serve as the genomic-based foundation for the overall phylogenetic relationships among genera of the family *Pasteurellaceae*. Based on this study’s results, we propose the following revised descriptions.

## Emended description of *Haemophilus* (Winslow et al., 1917)

Hae.mo’phi.lus. Gr. neut. n. *haîma*, blood; N. L. masc. Adj. suff. *-philus*, loving; from Gr. masc. Adj. *philos*, on; N. L. masc. n. *Haemophilus*, blood-lover.

Bacteria belonging to the family *Pasteurellaceae* (Holt et al., 1994; Kuhnert and Christensen, 2008). Gram-negative, non-motile, non-spore-forming, pleomorphic rods or coccobacilli, occasionally forming short filaments. Cell size ranges from 0.3–0.8 × 0.5–3 μm. Colonies on enriched media (e.g., chocolate agar) are smooth, convex, grayish and translucent; capsulated strains may appear mucoid or iridescent. *Haemophilus* species are aerobic, capnophilic, facultative anaerobes; some species can grow anaerobically under specific conditions. Some species require hemin or porphyrin (factor X) or NAD or NADP (factor V), or both, for growth; others need only NAD+. Growth requirements vary; some species need both X (hemin) and V (NAD) factors, while others can synthesize porphyrins and require only V factor. Catalase and oxidase reactions are positive; urease, indole and ornithine decarboxylase activities vary. β-Hemolysis is present in some species. Acid is produced from particular sugars (e.g., D-ribose, D-galactose, D-glucose), while fructose, mannose, mannitol, sorbitol and sucrose are typically not fermented. β-Galactosidase (ONPG) activity is negative. Member species colonize human mucosal surfaces, such as the nasopharynx, oral cavity and conjunctiva. The pathogenic potential ranges from harmless colonization to mild ocular or respiratory infections, with invasive disease linked to certain capsulated strains. The type species is *H. influenzae* (Lehmann and Neumann 1896; Winslow et al., 1917) (Approved Lists 1980).

*Average DNA G* + *C content (mol%)*: 38.0% (genome analysis).

*Median total length*: 1.85 Mb.

*Median protein count*: 1,713.

*Type strain*: ATCC 33391^T^ = CCUG 23945^T^ = CIP 102514^T^ = DSM 4690^T^ = NCTC 8143^T^.

*EMBL/GenBank/DDBJ accession number (16S rRNA gene)*: M35019.

*ENA/GenBank/DDBJ accession number (genome)*: GCF_001457655.1.

## Description of *Haemophilus quentini* sp. nov.

*Haemophilus quentini* (quen.ti’ni.); N. L. gen. n. *quentini,* of Quentin, named in honor of Roland Quentin for his substantial contributions to *Haemophilus influenzae* biotype IV research.

Small, pleomorphic rods or coccobacilli, occasionally forming short filaments. Colonies on chocolate agar are smooth, grayish, and translucent, usually non-hemolytic, with growth enhanced by 5–10% CO₂. Growth requires both X (hemin) and V (NAD) factors, and porphyrin synthesis from δ-aminolevulinic acid is absent. Catalase, oxidase, urease and ornithine decarboxylase tests are positive, while indole and *β*-galactosidase (ONPG) are negative. Nitrate is reduced and alkaline phosphatase is produced. Acid is produced from D-glucose, D-galactose, D-ribose and D-xylose, but not from D-fructose, D-mannose, D-mannitol, D-sorbitol, sucrose, lactose or other tested carbohydrates.

This species has been isolated from the urogenital tract of men with urethritis symptoms and from neonatal infections, including bacteremia and meningitis (Quentin et al., 1996). Identification is challenging and often requires molecular techniques, such as 16S rRNA gene sequence comparative analysis or multilocus sequence analysis (MLSA), as traditional phenotypic methods may not reliably distinguish it from other *Haemophilus* species (Mak et al., 2005).

*Average DNA G* + *C content (mol%)*: 38.5% (genome analysis).

*Median total length*: 2.15 Mb.

*Median protein count*: 2,198.

*Type strain*: MCCM 2026 ^T^ = CCUG 36167^T^.

*ENA/GenBank/DDBJ accession number (16S rRNA gene)*: AF224307.

*ENA/GenBank/DDBJ accession number (genome)*: GCF_001702075.1.

## Description of *Parahaemophilus* gen. nov.

*Parahaemophilus* (Pa.ra.hae.mo’phi.lus); Gr. prep. *para*, beside, alongside of, similar; N.L. masc. n. *Haemophilus*, a bacterial genus name; N.L. masc. n. *Parahaemophilus*, a genus resembling *Haemophilus*.

Small pleomorphic Gram-negative rods, sometimes coccoid or filamentous. Colonies on enriched media, such as chocolate agar, are usually grayish-white to yellowish, convex, and 1–2 mm in size after 24 h. β-Hemolysis may occur in some strains. Cells are NAD (V-factor) dependent but X-factor (hemin) independent, with porphyrins produced from δ-aminolevulinic acid. Oxidase and catalase activities are usually positive, although some strains may exhibit weak reactions. Growth is not significantly enhanced by CO₂.

The genus shows limited carbohydrate use, producing acid from a small list of sugars, including, D-mannose, D-fructose, D-galactose, D-glucose and sometimes maltose and sucrose. Enzymatic activity varies for urease, indole, ornithine decarboxylase and β-galactosidase (ONPG), while α- and β-glucosidases, β-xylosidase, α-fucosidase and nitrate reduction are usually negative. Phosphatases and leucine arylamidase activities may be present.

Members are commensal in the human oral cavity and pharynx, although some can colonize the genital tract or other sterile sites. They function as opportunistic pathogens, occasionally causing bloodstream infections, endocarditis and biliary tract infections. The type species is *P. parainfluenzae* comb. nov. (Rivers, 1922) (Approved Lists 1980).

*Average DNA G* + *C content (mol%)*: 39.6% (genome analysis).

*Median total length*: 2.06 Mb.

*Median protein count*: 1,943.

*Type strain*: ATCC 33392^T^ = CCUG 12836^T^ = CIP 102513^T^ = DSM 8978^T^ = NCTC 7857^T^.

*ENA/GenBank/DDBJ accession number (16S rRNA gene)*: AY362908.

*ENA/GenBank/DDBJ accession number (genome)*: GCF_900450845.1.

## Description of *Parahaemophilus parainfluenzae* comb. nov.

*Parahaemophilus parainfluenzae* (pa.ra.in.flu.en’zae) Gr. prep. *para*, beside, alongside of, similar; N.L. gen. n. *influenzae*, specific epithet (‘of influenza’); N.L. gen. n. *parainfluenzae*, resembling (*Haemophilus*) *influenzae*.

Basonym: *Haemophilus parainfluenzae* (Rivers, 1922) (Approved Lists 1980).

Small pleomorphic Gram-negative rods, occasionally coccoid or filamentous in shape. Colonies on chocolate agar are grayish-white or sometimes yellowish, opaque and grow to a diameter of 1–2 mm after 24 h. Strains with abnormal colony shapes, such as serrated edges, rough-wrinkled colonies, or granular appearance, are not uncommon. Irregular colonies are typically cohesive in texture and can be slid intact across the agar surface. β-hemolysis varies and is observed in some strains. Expression of tryptophanase, urease and ornithine decarboxylase varies, similar to *H. influenzae*, and has been used for biotyping.

Encapsulation of *P. parainfluenzae* has been reported, with 7% of non-hemolytic, V-factor-dependent strains testing positive for capsule. Encapsulated strains produce smaller, smooth colonies that are evenly suspended in saline and cannot be moved intact across the agar surface. Iridescence on translucent media is bright, and these strains do not agglutinate with *H. influenzae* typing sera. The genetic basis for encapsulation involves capsular transport regions I and III, similar to *H. influenzae*, while the serotype-specific genes A–D are unique.

NAD (V-factor) dependent, X-factor (hemin) independent and porphyrin-positive from δ-aminolevulinic acid. Catalase and oxidase tests are positive. Urease, indole and ornithine decarboxylase activities vary. Methyl red, Voges–Proskauer, alkaline phosphatase, *α*-fucosidase, nitrate reduction, alanine aminopeptidase, phosphatase and esterase are negative. β-Galactosidase (ONPG) activity is variable, while α- and β-glucosidase, β-xylosidase and α-galactosidase are usually negative. Acid production occurs from D-mannose, D-fructose, D-galactose and D-glucose but not from L-arabinose, D-arabinose, D-xylose, D-mannitol, D-sorbitol, L-fucose, D-ribose, or most other sugars. Growth is not enhanced by CO₂.

Clinically, *P. parainfluenzae* is a common commensal of the human oral cavity and pharynx and can colonize the genital tract. It is considered an opportunistic pathogen and is linked to infective endocarditis, i.e., as part of the HACEK group, bacteremia, urinary tract infections and, occasionally, respiratory tract infections. Genital carriage has been reported in pregnant women and men with acute urethritis. Its role in human disease is often underestimated because of its fastidious growth requirements and frequent presence as a commensal.

*Average DNA G* + *C content (mol%)*: 39.4% (genome analysis).

*Median total length*: 2.05 Mb.

*Median protein count*: 1,934.

*Type strain*: ATCC 33392^T^ = CCUG 12836^T^ = CIP 102513^T^ = DSM 8978^T^ = NCTC 7857^T^.

*ENA/GenBank/DDBJ accession number (16S rRNA gene)*: AY362908.

*ENA/GenBank/DDBJ accession number (genome)*: GCF_900450845.1.

## Description of *Parahaemophilus pittmaniae* comb. nov.

*Parahaemophilus pittmaniae* (pitt.ma’ni.ae); N. L. gen. n. *pittmaniae*, of Pittman, named in honor of Margaret Pittman for her substantial contributions to *Haemophilus* research.

Basonym: *Haemophilus pittmaniae* (Nørskov-Lauritsen et al., 2005).

Small pleomorphic gram-negative rods, sometimes forming filamentous cells. Colonies on chocolate agar are convex, grayish-white and grow to 1–2 mm in diameter after 24 h. β-Hemolysis occurs on horse- or sheep blood agar. Growth depends on NAD (V-factor) but is independent of X-factor (hemin), with porphyrins produced from δ-aminolevulinic acid. Oxidase and catalase reactions are negative or weakly positive. Growth is not significantly enhanced by CO₂.

The organism does not produce urease, indole, ornithine decarboxylase, α-fucosidase, or nitrate reduction. It tests positive for alkaline phosphatase, acid phosphatase, and alanine aminopeptidase. Esterase activity is negative. β-galactosidase (ONPG) is positive, while α-and β-glucosidase, β-xylosidase and α-galactosidase are generally negative.

Carbohydrate fermentation is limited. Acid production is positive for D-mannose, D-fructose, D-galactose, D-glucose, maltose and sucrose, but negative for L-arabinose, D-arabinose, D-xylose, D-mannitol, D-sorbitol, L-fucose and D-ribose.

Phylogenetically, it is closely related to *P. parainfluenzae* based on 16S rRNA and housekeeping gene sequence comparisons, DNA–DNA hybridization and genome sequence Average Nucleotide Identity (ANI).

Clinically, it is part of the normal human oral microbiota, with the type strain isolated from saliva. It can serve as an opportunistic pathogen and has been recovered from infections of the bloodstream, bile, and other sterile sites.

*Average DNA G* + *C content (mol%)*: 42.4% (genome analysis).

*Median total length*: 2.20 Mb.

*Median protein count*: 1,943.

*Type strain*: HK 85^T^ = CCUG 48703^T^ = DSM 21203^T^ = NCTC 13334^T^.

*ENA/GenBank/DDBJ accession number (16S rRNA gene)*: AJ290755.

*ENA/GenBank/DDBJ accession number (genome)*: GCF_900186995.1.

## Description of *Pseudohaemophilus* gen. nov.

*Pseudohaemophilus* (Pseu.do.hae.mo’phi.lus); Gr. masc. adj. *pseudês*, false; N.L. masc. n. *Haemophilus*, a bacterial genus name; N.L. masc. n. *Pseudohaemophilus*, a false *Haemophilus*.

Small Gram-negative rods, non-motile, occasionally coccoid or filamentous in shape. Colonies on chocolate agar are smooth, convex and opaque; on blood agar, *β*-hemolysis is variable or absent. Growth requires V factor (NAD) but not X factor (hemin), as porphyrins can be synthesized from *δ*-aminolevulinic acid. Growth in enriched CO₂ atmospheres varies among species.

Members of the genus are primarily isolated from humans and can be linked to respiratory infections, acute pharyngitis, dental alveolitis, or infections in cystic fibrosis patients. The type species is *P. parahaemolyticus* comb. nov. (Pittman, 1953) (Approved Lists 1980).

*Average DNA G* + *C content (mol%)*: 40.1% (genome analysis).

*Median total length*: 2.14 Mb.

*Median protein count*: 2,041.

*Type strain*: ATCC 10014^T^ = CCUG 3716^T^ = CIP 56.86^T^ = DSM 21417^T^ = NCTC 8479^T^.

*ENA/GenBank/DDBJ accession number (16S rRNA gene)*: AJ295746.

*ENA/GenBank/DDBJ accession number (genome)*: GCF_900450675.1.

## Description of *Pseudohaemophilus parahaemolyticus* comb. nov.

*Pseudohaemophilus parahaemolyticus* (pa.ra.hae.mo.ly’ti.cus); Gr. prep. *Para*, besides, alongside of, near, like N.L. masc. adj. *haemolyticus*, specific epithet (‘hemolytic); N. L. masc. Adj. *parahaemolyticus*, resembling (*Haemophilus*) *haemolyticus*.

Basonym: *Haemophilus parahaemolyticus* (Pittman, 1953) (Approved Lists 1980).

A small, regular Gram-negative rod with occasional filamentous forms. Colonies on chocolate agar resemble those of the smooth type of *H. parainfluenzae*, while a distinct β-hemolytic zone appears when cultured on blood agar. The organism is NAD (V-factor) dependent, does not need X-factor and is porphyrin-positive from δ-aminolevulinic acid. Biochemically, it tests positive for catalase, oxidase and urease, but negative for indole and β-galactosidase (ONPG), and it does not grow better in CO₂. Colonies are larger and less translucent than those of related species and, in broth, they form stringy, floccular sediment with a clear supernatant.

Carbohydrate utilization is limited; fermentation is positive for glucose, fructose and galactose (delayed), but negative for mannitol, sorbitol, xylose, ribose and most other sugars. The organism also produces an IgA1 endopeptidase capable of cleaving human IgA1. Clinically, it is most often associated with acute pharyngitis.

*Average DNA G* + *C content (mol%)*: 40.1% (genome analysis).

*Median total length*: 2.08 Mb.

*Median protein count*: 2,008.

*Type strain*: ATCC 10014 ^T^ = CCUG 3716 ^T^ = CIP 56.86 ^T^ = DSM 21417^T^ = NCTC 8479 ^T^.

*ENA/GenBank/DDBJ accession number (16S rRNA gene)*: AJ295746.

*ENA/GenBank/DDBJ accession number (genome)*: GCF_900450675.1.

## Description of *Pseudohaemophilus paraphrohaemolyticus* comb. nov.

*Pseudohaemophilus paraphrohaemolyticus* (par.a.phro.hae.mo.ly’ti.cus); Gr. prep. *Para*, beside, near, similar; N. L. *aphro-*, abbreviation of the specific epithet aphrophilus; Gr. neut. n. *haîma*, blood; N. L. masc. Adj. *lyticus*, able to dissolve; from Gr. masc. Adj. *lytikos*, dissolving; N. L. masc. Adj. *paraphrohaemolyticus*, intended to mean similar to *Haemophilus aphrophilus*, but hemolytic.

Basonym: *Haemophilus paraphrohaemolyticus* (Zinnemann et al., 1971) (Approved Lists 1980).

Small Gram-negative rods that are V-factor dependent but not X-factor dependent, favoring growth in elevated CO₂ and producing hemolysis on horse-blood agar. Colonies are generally larger and less translucent than related species and may exhibit aggregative growth in broth. The bacterium tests positive for porphyrin (δ-aminolevulinate). It produces β-hemolysis on blood agar but does not show increased growth with added CO₂. Colonies show variable catalase activity and are urease-positive.

Biochemically, it is distinguished from related hemolytic species by being β-galactosidase (ONPG) positive and lacking IgA1 protease activity. Other traits include ornithine decarboxylase negative, fermenting glucose and fructose, variable reactions with galactose and not utilizing xylose, mannitol, sorbitol, ribose or mannose.

*Average DNA G* + *C content (mol%)*: 40.8% (genome analysis).

*Median total length*: 2.04 Mb.

*Median protein count*: 1,955.

*Type strain*: ATCC 29237^T^ = CCUG 3718^T^ = CIP 102512^T^ = DSM 21451^T^ = NCTC 10670^T^.

*ENA/GenBank/DDBJ accession number (16S rRNA gene)*: M75076.

*ENA/GenBank/DDBJ accession number (genome)*: GCF_002015045.1.

## Description of *Pseudohaemophilus sputorum* comb. nov.

*Pseudohaemophilus sputorum* (spu.to’rum); L. neut. n. *sputum*, spit, sputum; L. gen. pl. n. *sputorum* of sputa.

Basonym: *Haemophilus sputorum* (Nørskov-Lauritsen et al., 2012).

Cells are non-motile, small, regular rods, measuring 0.3–0.5 μm by 2.0–3.0 μm, with occasional coccoid forms. Colonies on chocolate agar are convex, whitish, opaque and reach a diameter of 0.5–1.5 mm within 24 h. Zones of β-haemolysis form around colonies on horse or sheep blood agar; some strains are non-hemolytic and do not grow on blood agar without a feeder strain (CCUG 13788^T^ is hemolytic). Cells cannot synthesize nicotinamide adenine dinucleotide *de novo*, so growth depends on V factor. Porphyrins are produced from δ-aminolevulinic acid, meaning haemin (X factor) is not needed. Cells produce β-galactosidase, urease and leucine arylamidase but are negative for tryptophanase (indole test), arginine dihydrolase, lysine decarboxylase, ornithine decarboxylase and phenylalanine arylamidase. H_2_S is absent or weakly emitted (lead acetate test) and IgA1 protease is not produced. According to the VITEK-2 NH identification card, fermentation produces acid from d-glucose, fructose, d-maltose and maltotriose, but not from N-acetyl-β-d-glucosamine, d-xylose, d-ribose, d-mannose, lactose or d-malate. Variable fermentation occurs with d-galactose (CCUG 13788^T^ is negative). The oxidase (spot test) is positive and H_2_O_2_ is variably decomposed (CCUG 13788^T^ is negative). The species yields a distinctive MALDI-TOF mass spectrum that sets it apart from other recorded bacteria. *P. sputorum* is occasionally involved in human infections and has been isolated from blood, sputum of cystic fibrosis patients and tooth alveolitis (Nørskov-Lauritsen et al., 2012).

*Average DNA G* + *C content (mol%)*: 39.6% (genome analysis).

*Median total length*: 2.06 Mb.

*Median protein count*: 1,946.

*Type strain*: CCUG 13788 ^T^ = DSM 24472 ^T^.

*ENA/GenBank/DDBJ accession number (16S rRNA gene)*: JF506642.

*ENA/GenBank/DDBJ accession number (genome)*: GCF_000238795.1.

## Description of *Pseudohaemophilus minor* comb. nov.

*Pseudohaemophilus minor* (mi‘nor); L. comp. Masc. adj. *Minor*, less, smaller.

Basonym: *Actinobacillus minor* (Møller et al., 1996).

Cells are small Gram-negative rods, measuring 1.6–2.4 μm in length, with occasional slight variations in shape. They are non-motile, dependent on V-factor, independent of X-factor and non-hemolytic. Colonies on chocolate agar are smooth, grayish and about 0.8 mm in diameter after 48 h of incubation.

The species is catalase-negative, oxidase variable, urease-positive, and indole-negative. Lysine and ornithine are not decarboxylated and α-fucosidase activity is absent. The CAMP reaction is negative.

Acid is produced from glucose, D-mannose, maltose, lactose, sucrose and raffinose, while L-arabinose, D-arabinose, D-xylose, D-mannitol, D-sorbitol and other tested sugars are not fermented. β-galactosidase (ONPG) is positive, and other enzymatic activities, such as indole, α-fucosidase and decarboxylases, are negative.

*Average DNA G* + *C content (mol%)*: 39.7% (genome analysis).

*Median total length*: 2.28 Mb.

*Median protein count*: 2,127.

*Type strain*: NM 305 ^T^ = CCUG 38923 ^T^ = CIP105314 ^T^.

*ENA/GenBank/DDBJ accession number (16S rRNA gene)*: U65582.

*ENA/GenBank/DDBJ accession number (genome)*: GCF_000175195.1.

## Description of *Pseudactinobacillus* gen. nov.

*Pseudactinobacillus* (Pseud.ac.ti.no.ba.cil’lus. Gr. masc. adj. *pseudês*, false. N.L. masc. n. *Actinobacillus*, a bacterial genus name; N.L. masc. n. *Pseudactinobacillus*, false *Actinobacillus*, from the sporadic misdiagnosis of the type species as *Actinobacillus*.

Members of the genus include a human pathogen associated with genital ulcerative disease. No carrier state in healthy individuals has been documented. The description is as the species description of the only species of the genus. The type species of genus *Pseudactinobacillus* is *Pseudactinobacillus ducreyi* (Neveu-Lemaire, 1921).

*Average DNA G* + *C content (mol%)*: 38.2% (genome analysis).

*Median total length*: 1.65 Mb.

*Median protein count*: 1,452.

*Type strain*: ATCC 33940 ^T^ = CCUG 4438 ^T^ = CIP 54.2 ^T^ = DSM 8925 ^T^ = NCTC 10945 ^T^.

*ENA/GenBank/DDBJ accession number (16S rRNA gene)*: M63900.

*ENA/GenBank/DDBJ accession number (genome)*: GCF_900109315.1.

## Description of *Pseudactinobacillus ducreyi* comb. nov.

*Pseudactinobacillus ducreyi* (du.crey’i.) N.L. gen. n. *ducreyi*, of Ducrey, named after Ducrey, the bacteriologist who first isolated this organism *Pseudactinobacillus*.

Basonym: “*Coccobacillus ducreyi*” (Neveu-Lemaire, 1921).

Cells and colonies are as described for the genus. *P. ducreyi* is the etiological agent of chancroid (soft chancre), a human genital ulcerative disease endemic in parts of Africa, where it may account for as many as 10% of genital ulcers. No carrier state in healthy individuals has been documented.

Cells are slender Gram-negative rods, 0.5 × 1.5–2.0 μm, occurring in pairs or short chains. Growth on standard laboratory media is slow, requiring as many as 72 h to produce small, flat, smooth, grayish, translucent colonies. The organism is hemin-dependent but NAD-independent. Weak β-hemolysis may be observed on blood agar. Catalase is negative, oxidase is positive. Most conventional phenotypic tests are inert and the species is asaccharolytic.

*Average DNA G* + *C content (mol%)*: 38.2% (genome analysis).

*Median total length*: 1.65 Mb.

*Median protein count*: 1,452.

*Type strain*: ATCC 33940^T^ = CCUG 4438^T^ = CIP 54.2^T^ = DSM 8925^T^ = NCTC 10945^T^.

*ENA/GenBank/DDBJ accession number (16S rRNA gene)*: M63900.

*ENA/GenBank/DDBJ accession number (genome)*: GCF_900109315.1.

## Description of *Targowskia* gen. nov.

Tar.gow’ski.a. N.L. fem. n. *Targowskia*, to honor Stanislaw and Hanna Targowski, who isolated the type species of the genus from diseased rabbits.

*Targowskia paracuniculus* was originally isolated from the gastrointestinal tracts of diseased young rabbits (*Oryctolagus cuniculus*). The description is as the species description of the only species of the genus. The type species of *Targowskia* is *T. paracuniculus* (Targowski and Targowski, 1979).

*Average DNA G* + *C content (mol%)*: 45.5% (genome analysis).

*Median total length*: 2.05 Mb.

*Median protein count*: 1,920.

*Type strain*: ATCC 29986 ^T^ = CCUG 43573 ^T^ = CIP 107045 ^T^ = DSM 21452 ^T^.

*ENA/GenBank/DDBJ accession number (16S rRNA gene)*: M75061.

*ENA/GenBank/DDBJ accession number (genome)*: GCF_002015115.1.

## Description of *Targowskia paracuniculus* comb. nov.

*Targowskia paracuniculus* (pa.ra.cu.ni’cu.lus.) Gr. prep. *para*, beside, alongside of, near, like; L. masc. n. *cuniculus*, a rabbit; N.L. masc. n. *paracuniculus*, resembling *cuniculus*, originally coined in reference to an unpublished *taxon*.

Basonym: *Haemophilus paracuniculus* (Targowski and Targowski, 1979) (Approved Lists 1984).

The species was associated with mucoid enteritis, a highly fatal disease of juvenile rabbits of unknown etiology. Of the six strains initially recovered, five were lost before extended characterization, and the species has not been reported since.

Cells are Gram-stain-negative, non-motile coccobacilli. Growth occurs on chocolate agar and is enhanced by CO₂. Colonies are non-hemolytic on blood agar. The species is catalase- and oxidase-positive. Growth requires V factor (NAD), whereas X factor (hemin) is not required; porphyrin synthesis is positive. The *nadV* gene is absent. Urease and indole reactions are positive. Ornithine decarboxylase activity is present. β-hemolysis is not observed. β-galactosidase activity (ONPG) is positive. Acid is produced from D-fructose. Acid is not produced from D-xylose, D-mannitol, D-ribose or D-galactose. No gas is produced from carbohydrates.

*Average DNA G* + *C content (mol%)*: 45.5% (genome analysis).

*Median total length*: 2.05 Mb.

*Median protein count*: 1,920.

*Type strain*: ATCC 29986 ^T^ = CCUG 43573 ^T^ = CIP 107045 ^T^ = DSM 21452 ^T^.

*ENA/GenBank/DDBJ accession number (16S rRNA gene)*: M75061.

*ENA/GenBank/DDBJ accession number (genome)*: GCF_002015115.1.

## Description *Pasteurella felis* comb. nov.

*Pasteurella felis* (fe’lis), *L*. gen, n. *felis*, of a cat.

Basonym: *Haemophilus felis* (Inzana et al., 1992).

*Pasteurella felis* consists of small, regular Gram-negative rods measuring approximately 0.45–0.55 × 1.5–1.7 μm, although spherical cells and occasional filamentous forms may also occur. Growth is stimulated by increased CO₂ concentrations, although this requirement is typically lost after repeated subculturing. Colonies on chocolate agar are raised, smooth and reach 0.5–1 mm in diameter within 24 h. On brain heart infusion agar supplemented with NAD, colonies are usually adherent and display a characteristic yellow pigmentation.

The species has been isolated both from the lower respiratory tract of a cat with obstructive pulmonary disease and from the nasopharynx of healthy cats, suggesting a dual commensal–opportunistic lifestyle within felids.

*Average DNA G* + *C content (mol%)*: *40.0*% (genome analysis).

*Median total length*: 2.33 Mb.

*Median protein count*: 2,107.

*Type strain*: TI189^T^ = ATCC 49733^T^ = CCUG 31170^T^ = CIP 103890^T^ = DSM 21192^T^.

*ENA/GenBank/DDBJ accession number (16S rRNA gene)*: AF224292.

*ENA/GenBank/DDBJ accession number (genome)*: GCF_009910635.1.

## Data Availability

The original contributions presented in the study are included in the article/Supplementary material, further inquiries can be directed to the corresponding authors.

## Footnotes

1
www.lpsn.dsmz.de/genus/haemophilus

2
https://microbializer.tau.ac.il/

3
https://github.com/dparks1134/CompareM

4
https://www.ccug.se/documents/chemotyping/CFA.methodpubl.pdf

## Glossary

AAI: Average Amino acid Identity
ANI: Average Nucleotide Identity
ANIb: BLAST-based Average Nucleotide Identity
CAZymes: Characterized Carbohydrate-Active Enzymes
CCUG: Culture Collection University of Gothenburg
CFA-FAMEs: Cellular Fatty Acid-Fatty Acid Methyl Esters
dDDH: digital DNA–DNA Hybridization
ECL: Equivalent Chain Length
GGDC: Genome-to-Genome Distance Calculator
GH: Glycoside Hydrolase Family
G1: *H. influenzae sensu stricto* cluster
G2: *H. parainfluenzae* cluster
G3: *Pasteurella sensu stricto* cluster
G4: *H. sputorum* cluster
HGT: Horizontal gene transfer
HMM: Hidden Markov Model
iTOL: interactive Tree of Life
KO: KEGG orthology
LPSN: List of Prokaryotic names with Standing in Nomenclature
LOS: Lipooligosaccharide
MLSA: Multi-Locus Sequence Analysis
NAD: Nicotinamide Adenine Dinucleotide
NCBI: National Center for Biotechnology Information
NJ: Neighbor-Joining
ONPG: Ortho-Nitrophenyl β-D-Galactopyranoside or β-Galactosidase
OGRI: Overall Genome Relatedness Index
PNPG: PNPG P-Nitrophenyl α-D-Glucopyranoside or α-Glucosidase
POCP: Percentage of Conserved Proteins
VF: Virulence Factor
VFDB: Virulence Factor DataBase
WGS: Whole Genome Sequence.
